# The full annual carbon balance of a subtropical coniferous plantation is highly sensitive to autumn precipitation

**DOI:** 10.1038/s41598-017-10485-w

**Published:** 2017-08-30

**Authors:** Mingjie Xu, Huimin Wang, Xuefa Wen, Tao Zhang, Yuebao Di, Yidong Wang, Jianlei Wang, Chuanpeng Cheng, Wenjiang Zhang

**Affiliations:** 1 Key Laboratory of Ecosystem Network Observation and Modeling, Institute of Geographic Sciences and Natural Resources Research, Chinese Academy of Sciences, Beijing, 100101 China; 2 College of Agronomy, Shenyang Agricultural University, Shenyang, 110866 China; 3 University of Chinese Academy of Sciences, Beijing, 100049 China; 4 Tianjin Key Laboratory of Water Resources and Environment, Tianjin Normal University, Tianjin, 300387 China; 5 State Key Laboratory of Hydraulics and Mountain River Engineering, Sichuan University, Chengdu, 610065 China

## Abstract

Deep understanding of the effects of precipitation on carbon budgets is essential to assess the carbon balance accurately and can help predict potential variation within the global change context. Therefore, we addressed this issue by analyzing twelve years (2003–2014) of observations of carbon fluxes and their corresponding temperature and precipitation data in a subtropical coniferous plantation at the Qianyanzhou (QYZ) site, southern China. During the observation years, this coniferous ecosystem experienced four cold springs whose effects on the carbon budgets were relatively clear based on previous studies. To unravel the effects of temperature and precipitation, the effects of autumn precipitation were examined by grouping the data into two pools based on whether the years experienced cold springs. The results indicated that precipitation in autumn can accelerate the gross primary productivity (GPP) of the following year. Meanwhile, divergent effects of precipitation on ecosystem respiration (Re) were found. Autumn precipitation was found to enhance Re in normal years but the same regulation was not found in the cold-spring years. These results suggested that for long-term predictions of carbon balance in global climate change projections, the effects of precipitation must be considered to better constrain the uncertainties associated with the estimation.

## Introduction

Along with elevated air temperature, climate change is predicted to cause dramatic variability in the precipitation regime, changing not only the annual precipitation amount but also the seasonal distribution of precipitation, which combine to influence various processes of terrestrial ecosystems^1, 2^, especially in monsoon regions. The monsoon is an important natural driver of ecosystem carbon and water exchanges in Asia^3^. Monsoon advance and retreat time greatly affect precipitation variability and, probably, the carbon sequestration process of the terrestrial ecosystem. Because forests are considered to have great potential to sequester carbon^4, 5^, understanding the effects of climate change on the carbon exchange processes of forests is of great importance.

Forest carbon fluxes are sensitive to climatic perturbations, especially at the edges of the growing season, such as early spring and late autumn^6, 7^. The effects of changing temperatures in early spring have been relatively well studied^8^. However, the effects of changes in precipitation in a given sensitive period are still less well known. According to previous studies, the effects of temperature and precipitation were thought to vary in different types of forests and under distinct climatic conditions^9–13^. For temperate and boreal forests, air temperature is generally considered the most critical factor controlling carbon sequestration^14^. Higher temperatures in early spring, associated with a longer growing season, were often found to result in higher gross primary productivity (GPP)^15, 16^. Similar regulation was also found in subtropical coniferous forest^8^, although in this region, precipitation was regarded as the most important factor in regulating inter-annual variation^17^. However, in water-limited regions, the influences of drought have been widely studied, especially extreme climate events, and they were generally found to negatively impact GPP, ecosystem respiration (Re) and net ecosystem productivity (NEP)^18–20^. However, the effects of precipitation on carbon balance remain unclear.

Carbon fluxes of forest ecosystems normally exhibit great inter-annual variation^21, 22^, which creates uncertainty in carbon budget estimation^23^. In regulating the inter-annual variability of carbon fluxes, individual climatic factors play a limited role in either temperature or water-limited forests^24–26^. Empirical evidence suggests that the variability in carbon flux is directly influenced by inter-annual climatic variability and by its lag effects^27–29^. Based on long-term datasets, more attention should be paid to the joint effects of air temperature and precipitation and their lag effects. In temperate and boreal regions, late summer water availability together with spring air temperature has been reported to regulate the inter-annual variability in carbon balance of Canadian Douglas fir stands^30^. In subtropical regions, early spring temperature and late summer and autumn soil water content were also found to be major factors controlling the inter-annual variation in net carbon uptake^8, 31^. However, most studies have only considered the immediate impacts of environmental factors^21, 32^, whereas commonly occurring lag effects at long time scales have conventionally been ignored^26, 33, 34^.

Some studies have revealed that the lag effects of climatic drivers on carbon fluxes may be more important than their immediate effects^24, 35, 36^. For instance, spring temperatures not only have effects on spring carbon budgets, they can also greatly affect annual ecosystem productivity^8, 37, 38^. Thus, a non-equilibrium framework is necessary to study the impacts of temperature change on current or future forest carbon balance^34, 37^. Precipitation can generate lag effects in ecosystem CO_2_ exchange by altering LAI, canopy chemistry^39^ and the soil microbial community^40^. Teklemariam *et al*. reported stronger correlations between carbon fluxes and climatic factors in the previous year than those in the current year^35^. Therefore, the direct and lagged effects of climatic factors on carbon fluxes must be considered jointly^25, 35^. Some studies have proposed overwhelming effects of air temperature and precipitation in the spring and autumn, but few researchers have considered them together and linked them to precipitation lag effects.

Southern China is characterized by a humid monsoon climate and has large subtropical coniferous plantations, which account for 41% of the total subtropical forest area^41^. Thus, it is important to conduct research on the carbon cycles in this region. Twelve years (2003–2014) of continuous eddy-covariance measurements recorded at a subtropical coniferous forest site were analyzed. Based on previous carbon flux research, the effects of autumn precipitation on GPP and Re were studied. The objectives of this study were to: (1) characterize the effects of autumn precipitation on GPP and its lag effects; and (2) demonstrate the effects of autumn precipitation on Re and reveal its mechanism.

## Results

### Seasonal and inter-annual variation of environmental factors

**Figure 1:**
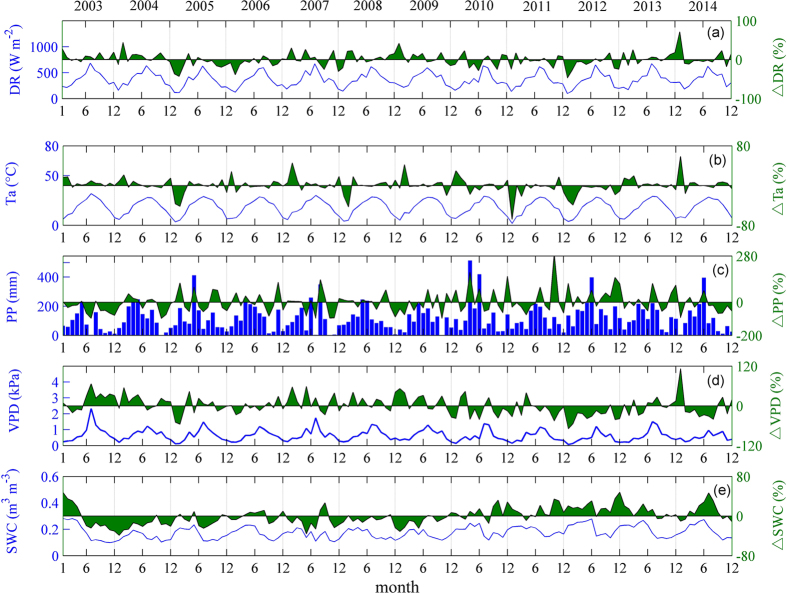
Multi-year (2003–2014) environmental conditions accumulated or averaged monthly on the left axis and monthly relative anomalies (%) of environmental factors on the right axis: (**a**) direct radiation (DR); (**b**) air temperature (Ta); (**c**) precipitation (PP); (**d**) vapor pressure deficit (VPD) and (**e**) soil water contents (SW) at 5 cm.

DR (direct solar radiation), Ta (air temperature), and VPD (vapor pressure deficit) values had a single peak and reached their maximums in July (Fig. 1a,b and d). The PP (precipitation) showed strong seasonal and inter-annual variation and was usually more in the first half year than that in the second half of the year (Fig. 1c). The trends in VPD and SWC (soil water contents) generally followed the seasonal course of Ta and precipitation (Fig. 1d and e).

**Figure 2:**
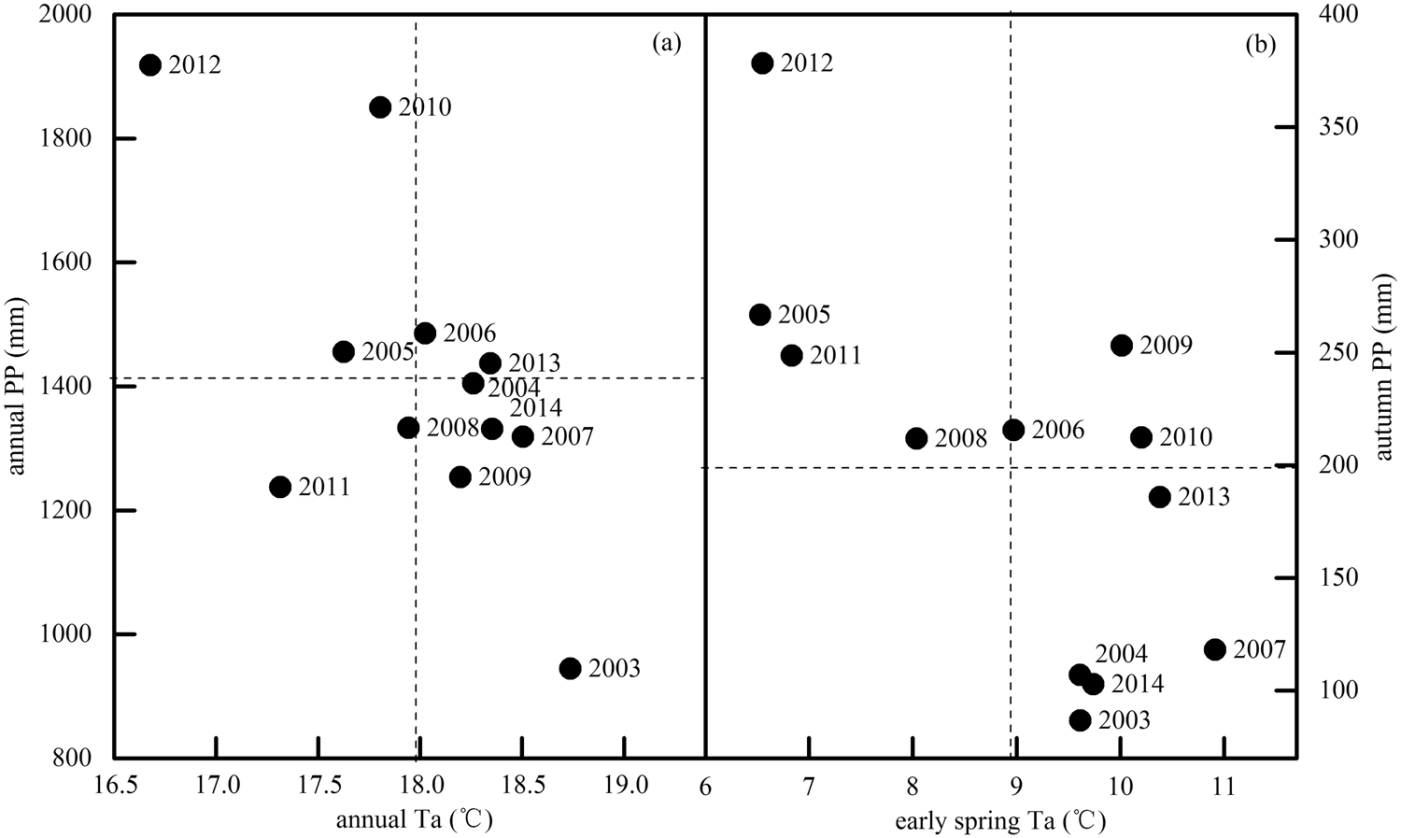
(**a**) Cumulative precipitation versus average air temperature for the whole year and (**b**) cumulative precipitation in autumn (September–November) versus average air temperature in early spring (January to March) during the period 2003–2014. Dotted lines represent the averages for the observed years.

The annual mean Ta from 2003 to 2014 ranged from 16.7 °C to 18.7 °C, with the minimum in 2012 and maximum in 2003, whereas the precipitation ranged from 944.9 mm in 2003 to 1918.3 mm in 2012 (Fig. 2a). In general, the precipitation decreased as the temperature increased at the annual scale. The Ta was lower in 2005, 2008, 2011 and 2012 than that in other years from January to March because cold early springs appeared in those years (Figs 1b and 2b). In normal years, the monthly mean temperatures were always above the active temperature threshold of 5 °C throughout the whole year. However, in the cold years of 2005, 2008, 2011 and 2012, the monthly average temperatures in January were 3.4 °C, 4.2 °C, 1.7 °C and 3.7 °C, respectively. In addition, because of the variation in the monsoon retreat time, the inter-annual variation in precipitation in autumn (from September to November) was large (Figs 1c and 2b). The coefficients of variation (CV) were 56.6%, 105.4% and 75.5% in September, October and November, respectively. The precipitation in the autumn of 2003, 2004, 2007, 2013 and 2014 was respectively 56.4%, 46.3%, 40.6%, 6.6% and 48.3% lower than multi-year average.

### Seasonal and inter-annual variation of carbon fluxes

**Figure 3:**
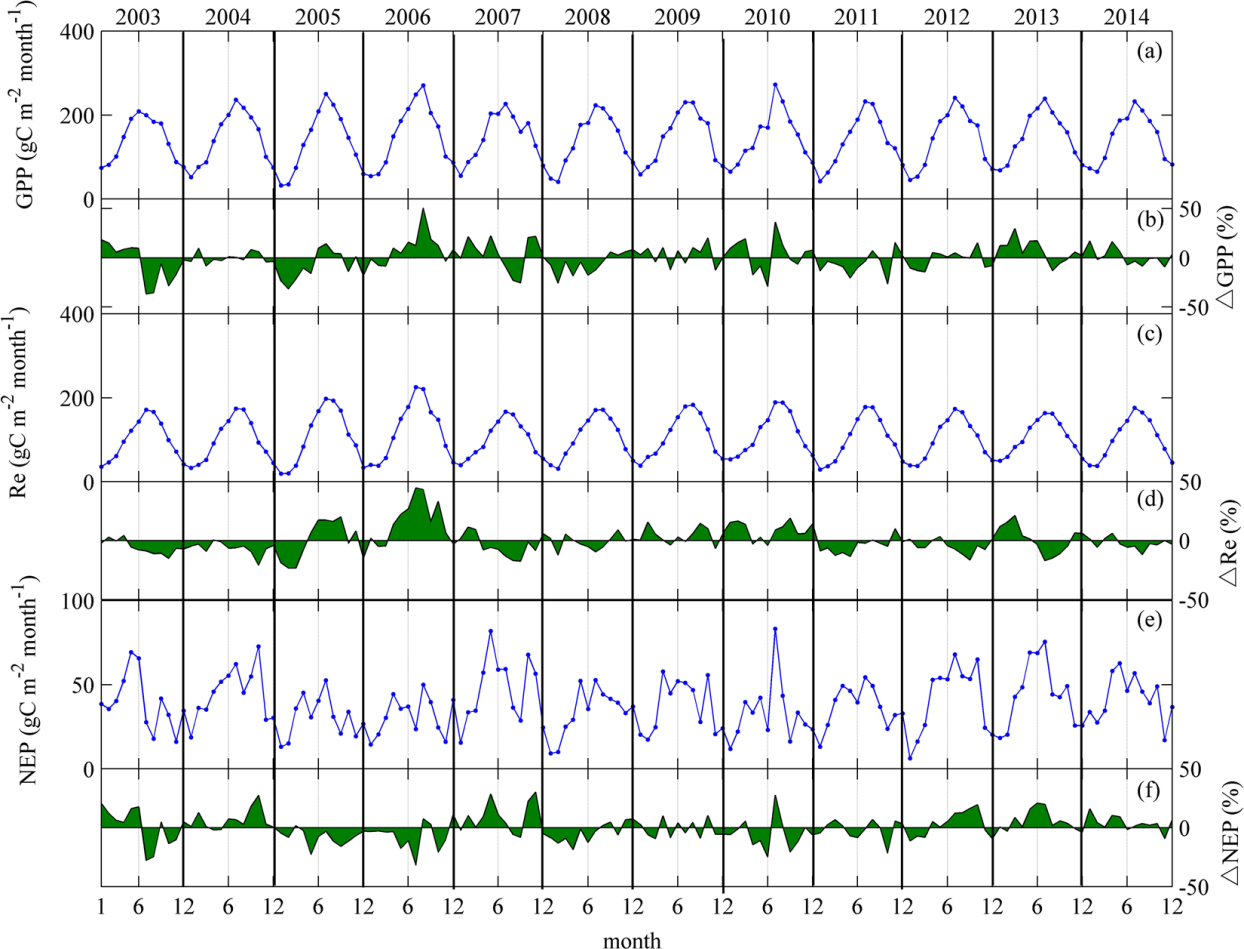
Multi-year (2003–2014) carbon fluxes averaged monthly on the left axis and their monthly relative anomalies (%) on the right axis: (**a**,**b**) gross primary productivity (GPP); (**c**,**d**) ecosystem respiration (Re) and (**e**,**f**) net ecosystem productivity (NEP).

The GPP and Re exhibited clear seasonal variation, with peaks in July in most observed years, whereas the seasonal variation in NEP was more complex because of its dependence on both GPP and Re (Fig. 3). The multi-year monthly average of NEP also peaked in July with a value of 55.6 ± 16.4 gC m^−2^ month^−1^.

**Figure 4:**
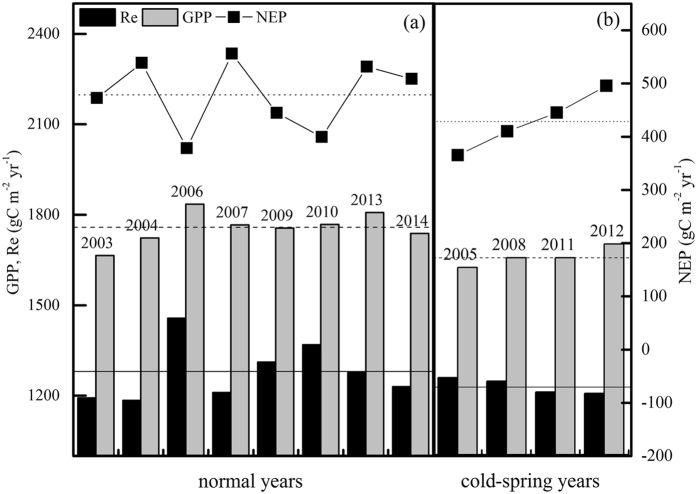
Yearly values of cumulative carbon fluxes calculated. The values were separated into two groups according to whether the year experienced a cold spring. The dashed, solid and dotted lines represent the average GPP, Re and NEP over the observed years, respectively.

Over the twelve years, the average annual GPP was 1724.0 ± 63.8 gC m^−2^ yr^−1^ (CV = 3.7%), with a maximum of 1835.5 gC m^−2^ yr^−1^ in 2006 and a minimum of 1621.5 gC m^−2^ yr^−1^ in 2005 (Fig. 4). The annual Re ranged from 1184.6 gC m^−2^ yr^−1^ in 2004 to 1457.5 gC m^−2^ yr^−1^ in 2006, with an average of 1262.2 ± 78.2 gC m^−2^ yr^−1^ (CV = 6.2%). As the difference between GPP and Re, the multi-year average NEP was 461.8 ± 62.2 gC m^−2^ yr^−1^. The CV of NEP was 13.5%, which was a bit larger than that of GPP and Re.

The mean annual GPP in the years with cold early springs was significantly lower than that of the other years (*P* < 0.01). The average annual Re and NEP in the years with cold early springs were also lower than those of the other years, but the differences were statistical insignificant (*P* = 0.33 for Re, *P* = 0.23 for NEP) (Fig. 4). The anomalies in GPP in the years with cold springs were negative when the temperature anomalies were negative (Fig. 3b), but the same regulation was not found in the variation in Re.

To exclude the effects of cold springs, the annual carbon fluxes were separated into two groups (Fig. 4). In the normal years (2006, 2013, 2010, 2007 and 2009), the GPP was higher than the average. As shown in Fig. 2b, the autumn precipitation in the previous years, 2005, 2012, 2009, 2006 and 2008, respectively, was also above average. Among the years with cold springs, the GPP was higher only in 2012, and the precipitation was higher in 2011.

To compare the effects of autumn precipitation, we divided the observations into two groups, as shown in Fig. 4. In combination with Figs 2 and 3, the variation in the regulation of Re was found to follow the inter-annual variation in precipitation in normal years, with the opposite pattern in cold-spring years.

### The response of GPP to autumn precipitation in the previous year

**Figure 5:**
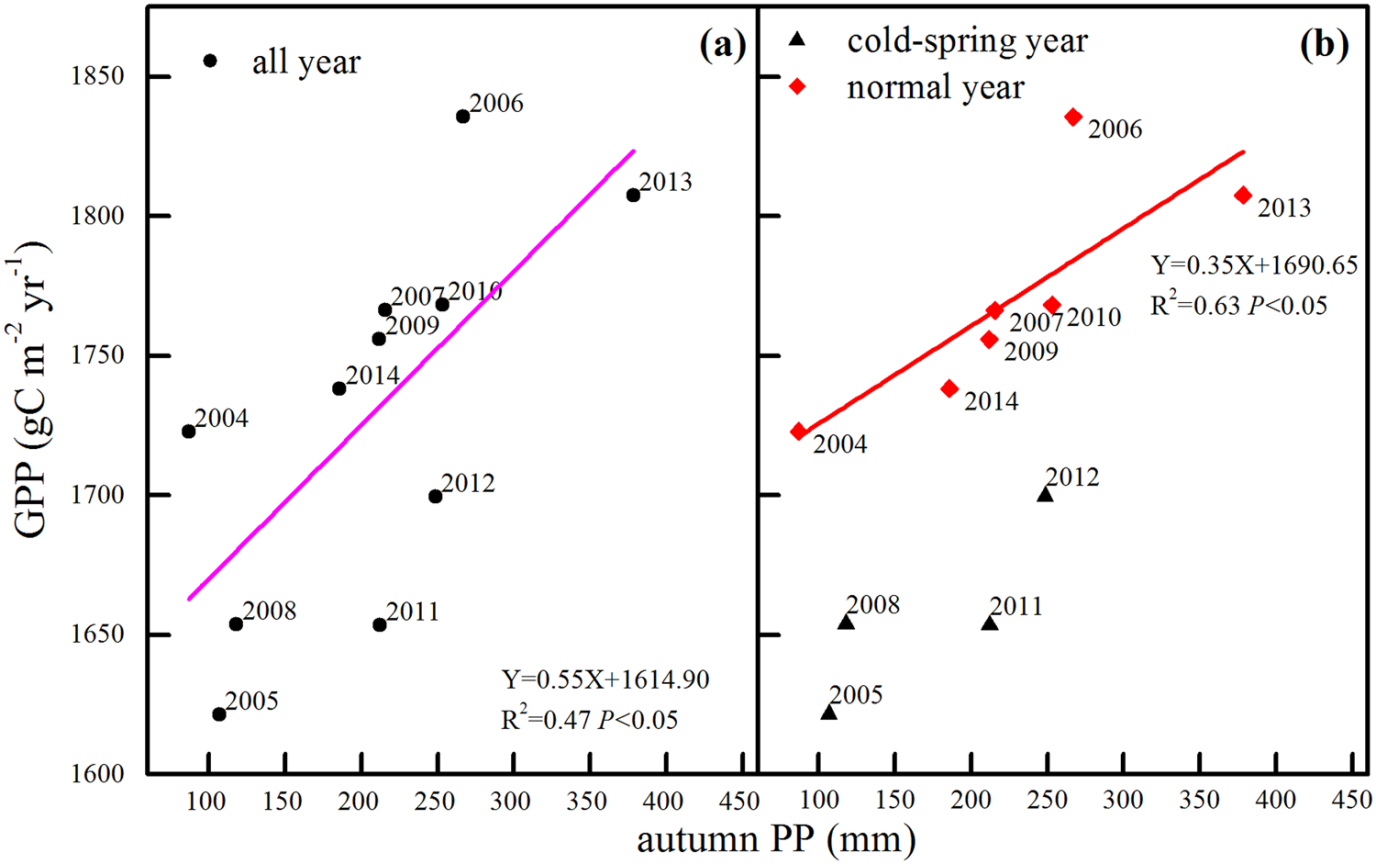
Relationships between the precipitation in the previous autumn and the annual GPP of the given year in 2003–2014: (**a**) all data grouped together and (**b**) data separated into two groups according to whether the year experienced a cold spring.

During the observed years, the annual GPP was found to be positively related to the autumn precipitation of the previous year (Fig. 5). When the data were placed into one group, the linear-fit relation seemed to be comparatively weak (Fig. 5a). The observed values were somewhat scattered around the fit line. In addition, the residual sum of squares was large.

When the data were classified by early spring cold, the autumn precipitation more clearly explained the annual GPP: 63% and 69% of GPP can be explained by the precipitation in the previous autumn in the years without and with cold early springs, respectively (Fig. 5b). The response slope of GPP to the precipitation in the previous autumn in the cold-spring years was greater than that of the ordinary years, which indicated a more sensitive response.

### The effects of autumn precipitation on Re

**Figure 6:**
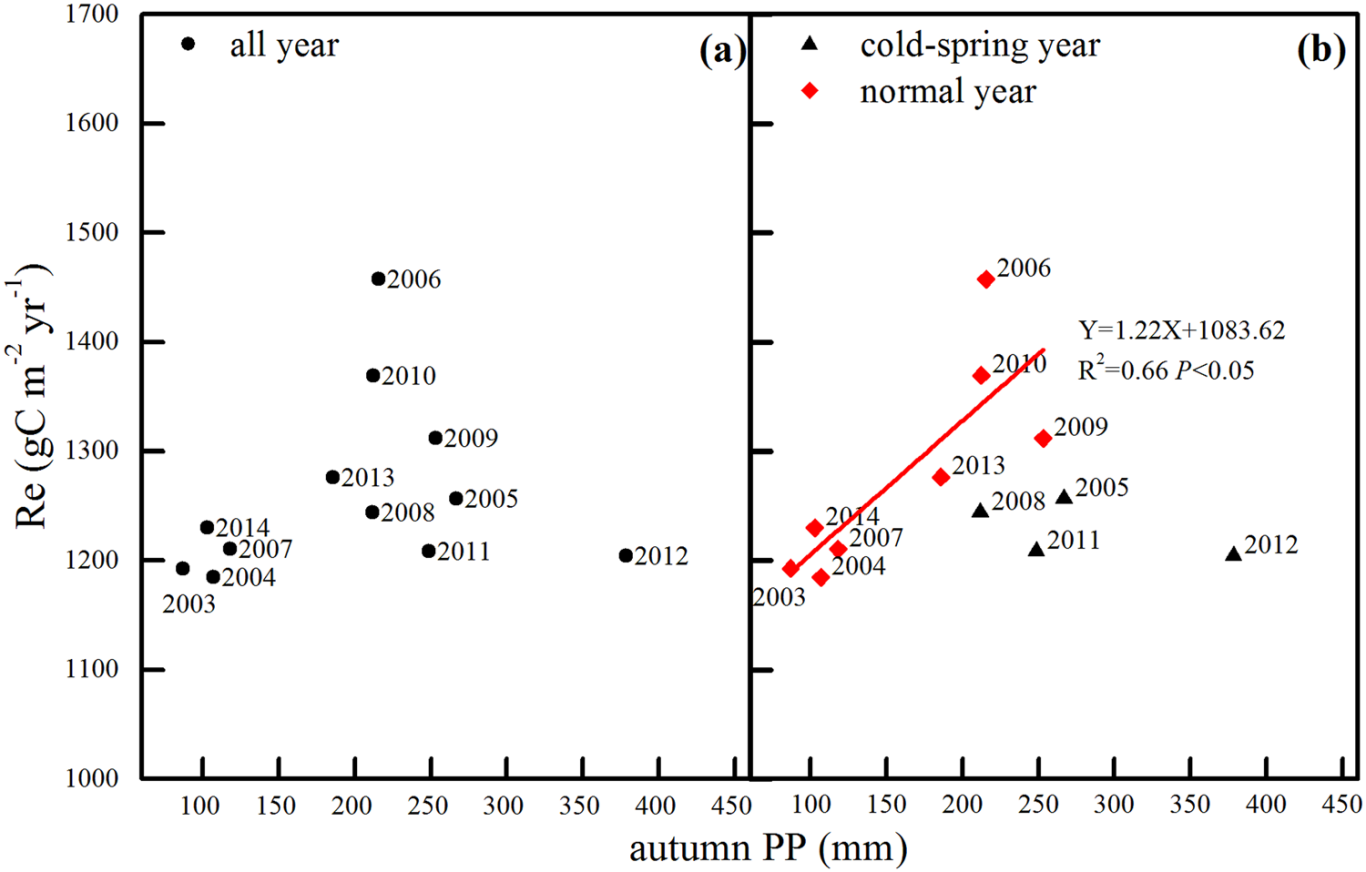
Relationships between autumn precipitation and annual Re in 2003–2014: (**a**) all data grouped together and (**b**) data separated into two groups according to whether the year experienced a cold spring.

Autumn precipitation had divergent effects on annual Re. When all of the annual Re values from 2003 to 2014 were studied, no clear relation was observed between Re and autumn precipitation (Fig. 6a). When the data were put into two pools, the effects of autumn precipitation emerged.

In the years without cold early springs, Re increased with increasing autumn precipitation. The liner relation was significant, and the R^2^ was 0.66. In the years with cold springs, Re showed decrease trend as autumn precipitation increased. The relation was not significant because of the limited samples, but autumn precipitation explained 43% of the variation in Re.

## Discussion

### Effects of temperature and precipitation on carbon fluxes

Over a long time, an ecosystem carbon budget will reach a dynamic equilibrium through the process of photosynthesis and respiration. As the variation in NEP depends on GPP and Re^42^, the controlling mechanisms of both GPP and Re should be examined to gain a comprehensive understanding of carbon budgets.

In addition, long-term observation is necessary to investigate the effects of climatic factors to avoid sampling in the trough or crest of a multi-annual pattern^28^. Therefore, we used 12-year observations to examine the mechanisms through which Ta and precipitation control GPP and Re in our study. During the observation years, the dataset represented much of the typical inter-annual variability at this site (Figs 3 and 4), including both typical and extreme climatic events; for example, the cold-spring years^8^, the typical El Niño event, which resulted in an extreme summer drought in 2003^17^, and a strong La Niña event that happened in 2009–2010^41^.

Previous studies have confirmed that variations in GPP and Re were driven by environmental forcing, especially temperature and precipitation^43, 44^. In the climate change context, warming temperatures and an altered precipitation regime would largely impact carbon budgets^45, 46^. Hence, many studies have focused on the effects of Ta and Precipitation. However, to date, no consensus has been reached regarding whether Ta or precipitation was the dominant factor^8, 47^. Especially at large temporal and spatial scales, the controlling mechanisms of Ta and precipitation remain controversial. Therefore, the existing models, which were constructed based on the driving effects of climatic factors, were inadequate to assess the inter-annual variability of carbon budgets^48^. Fortunately, when studies focused on a single site or a region, some instructive results were obtained^49^. These studies were conducted based on annual carbon budgets and their corresponding Ta and precipitation and reached general conclusions that temperate forests were temperature limited^12^. However, for subtropical and tropical forests, precipitation was more important than temperature^50^. At our study site, previous studies had determined that GPP, Re and NEP was depressed by summer-autumn drought^17^, whereas Zhang *et al*. indicated that the low temperature in spring played a more important role in regulating the inter-annual variability of annual net carbon uptake at this subtropical site^8^.

These results revealed that the full annual precipitation or annual mean Ta played limited roles in interpreting the variability in carbon budgets and their controlling mechanisms. Based on previous studies, the Ta and precipitation may be more crucial in transitional periods in the growing season, such as spring and autumn^6, 51, 52^. In other words, carbon budgets would be sensitive to Ta and/or precipitation within a given period^30^. In this study area, Ta in spring, especially low temperatures, could regulate phenology and depress carbon sequestration^8, 41^. Another key factor might be autumn precipitation because the carbon budget is easily affected by seasonal droughts^17, 31^. In addition, the autumn precipitation in this region is clearly affected by El Niño-Southern Oscillation (ENSO)^53^, with relatively great variability. Therefore, spring temperatures and autumn precipitation should be considered key factors in our study (Fig. 2b).

The effects of Ta and precipitation are always entangled. Therefore, to isolate the effects of autumn precipitation, we grouped the observed data into two pools according to whether the years experienced cold springs (Figs 4,5 and 6). The GPP and Re in cold-spring years were less than in normal years, which was consistent with the results of Zhang *et al*.^8^. Based on this result, we examined the contributions of autumn precipitation to GPP and Re in the two groups. The results underscored the prominent role that precipitation plays in the inter-annual variability of GPP and Re (Figs 4 and 5).

Previous studies have indicated that seasonal droughts occurred in this region in 2003 and 2007 and have observed the different responses of GPP and Re to water condition. At short-time scales, increased precipitation would be accompanied by a decrease in temperature and radiation, which would depress the photosynthesis of trees and thereby reduce GPP. However, at annual or longer time scales, the temperature and radiation are both generally high in this subtropical region. By contrast, the asynchronism of precipitation and temperature causes water to be a limiting factor, thereby reduced GPP and Re to different extents^17^. Therefore, a comprehensive understanding of the controlling mechanisms of precipitation was needed.

Recently, time-lag effects of precipitation have been reported in many studies^54–56^. Our study also found that GPP increased as autumn precipitation increased in the previous year for either normal or cold-spring years, and thus a time-lag effect of precipitation on GPP emerged. In contrast, for Re, previous studies mainly focused on the accelerating effects of precipitation on soil respiration and devoted less attention to the trade-off between depressed plant respiration and increased soil respiration. According to our results, precipitation showed varying effects on Re in normal and cold-spring years. Below, we discuss the lag effects of precipitation on GPP and the effects of precipitation on Re.

### The lagged effects of precipitation on variation in GPP

**Figure 7:**
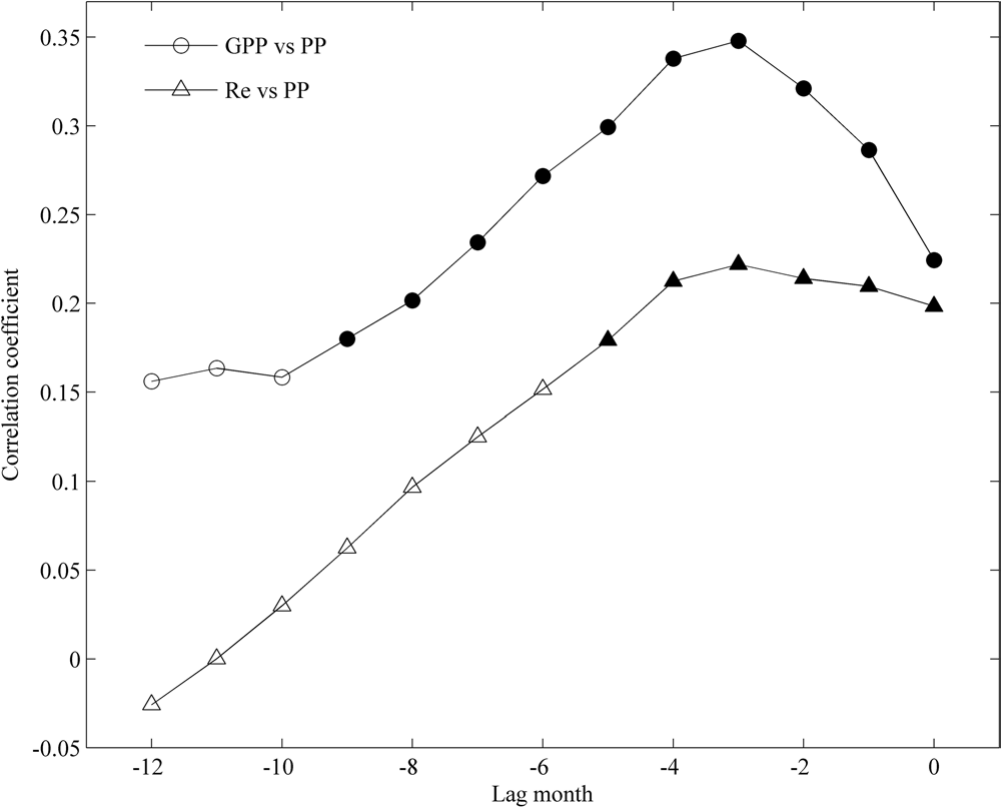
Correlation coefficients between annual GPP, Re and Precipitation (PP). Correlograms were calculated by shifting the precipitation backward one month at a time. The open signs represent coefficients that are not significant, and the closed signs indicate significant coefficients (p < 0.05).

Any individual climatic factor is far from adequate to explain carbon flux variability, especially precipitation, which has different controlling effects on GPP and Re. Our analysis shows that the precipitation in autumn will affect the Re of the given year and the GPP of the following year. Additionally, we used a statistical method to reveal and quantify the time lag effects of precipitation on ecosystem GPP and Re (Fig. 7). The statistical results strongly supported our analysis. Precipitation exhibited a significant 3–5 month lag effect on GPP (R = 0.51, *P* < 0.01) and a weak lag effect on Re in this subtropical coniferous plantation (Fig. 7).

Some studies have previously captured the lag effects of precipitation^57^, which are highly variable across different ecosystems and regions. Similar to our observation that annual GPP was sensitive to the precipitation of the previous autumn, Vasconcelos *et al*.^56^ found that aboveground net primary productivity in tropical forest regrowth increased following wetter dry-seasons. In contrast, Zhang *et al*. found weak lag effects of precipitation on GPP and Re in grassland ecosystems^31^. These different responses of ecosystems may be due to the different water use strategies of grasses, crops and trees. Yang *et al*. have reported that crops only use shallow soil water^58^, whereas trees can use deep soil water, especially in the dry season^59^.

The time-lag effects of precipitation in forests can also be ascribed to the altered water use strategies of plants under soil water stress^18^ or to the long turnover time of deep soil moisture^35^. Autumn precipitation is important to the recharge of soil water. Inadequate autumn precipitation can occasionally result in severe drought, in which decreases in GPP, Re and energy reserves would constrain the bud preformation in succeeding years^60, 61^. Similar results have been reported by Breda *et al*.^62^, where reduced net primary productivity was caused by reduced storage of carbohydrate, lipid and protein reserves during the previous drought year. Additionally, the altered water use strategy will increase deep soil water consumption, thereby leaving less water available for twig shooting in the following spring^41, 59^.

### Divergent responses of Re to autumn precipitation in normal and cold-spring years

Ecosystem respiration is a complex process that can be separated into two major components: plant respiration (root respiration was excluded) and soil respiration (Rs). Plant respiration includes leaf respiration, stem respiration, and respiration of the understory. This component is closely related to the GPP of the ecosystem^63, 64^ because they are coupled processes that are joined at the stomata. Therefore, the ratio between plant respiration and GPP would be a relatively constant value. The other important component of Re is soil respiration, which has been widely studied^65–67^. Soil respiration is generally considered to be controlled by temperature and sometimes by soil moisture and was thus deemed to be sensitive to precipitation^68, 69^. At our study site, soil respiration was also positively related to soil moisture and precipitation^70^.

**Figure 8:**
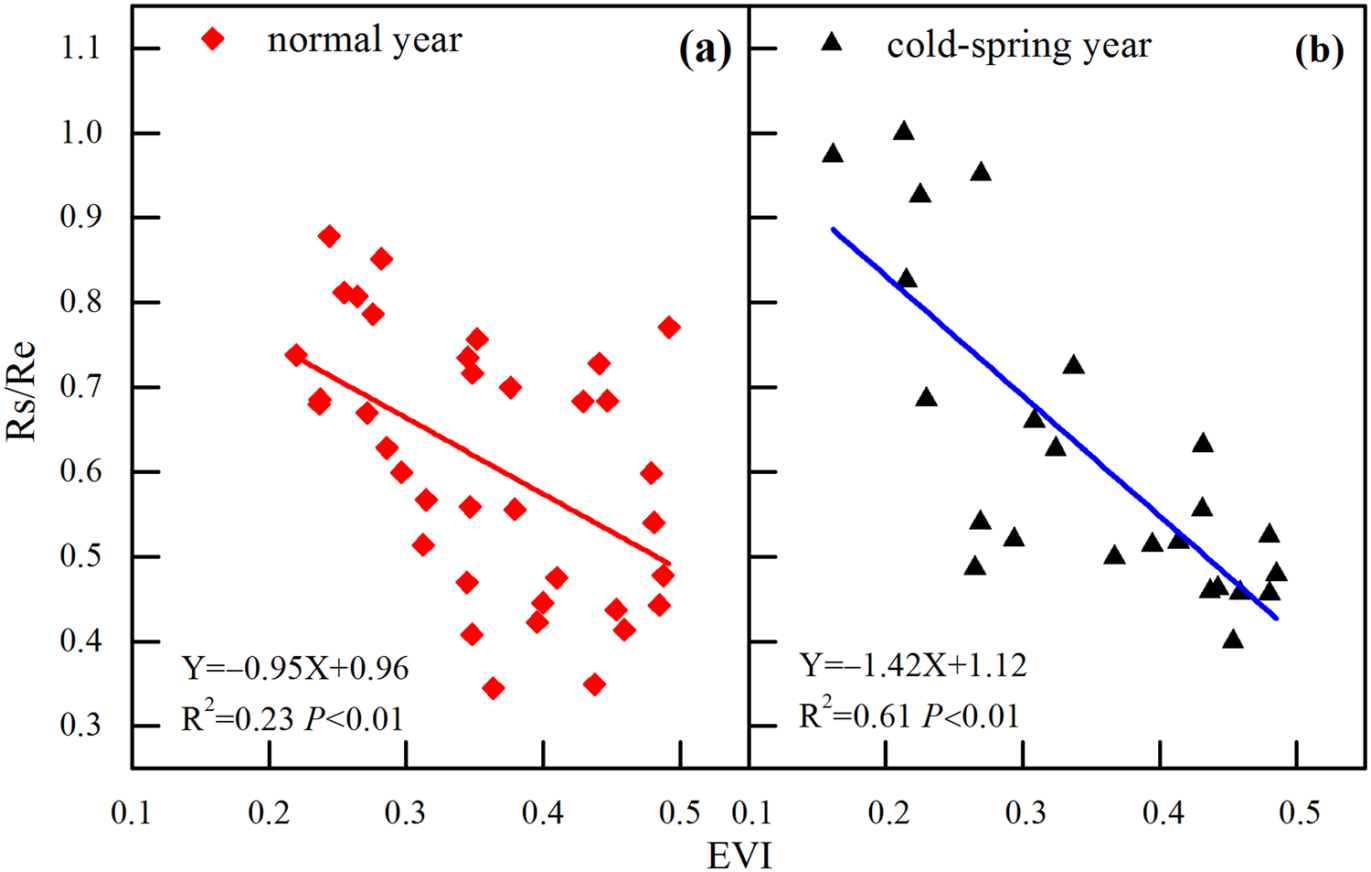
Relationship between the average monthly MODIS EVI and the ratios between soil respiration (Rs) and ecosystem respiration (Re): (**a**) for normal years and (**b**) for cold-spring years.

To conduct a comprehensive study of how precipitation affects Re, we should understand how precipitation affects these two components. Plant respiration was found to be positively related to air temperature, with obvious seasonal variation^71^. Precipitation events were always accompanied by relatively lower temperature and radiation, likely decreasing plant respiration. In contrast, soil respiration would be promoted by precipitation, as discussed by Wang *et al*.^70^. Therefore, to predict how precipitation would affect Re, we should consider the ratio between Rs and Re. At our study site, the Rs/Re ratio was negatively related to the enhanced vegetation index (EVI) (Fig. 8), with slopes of −0.95 in normal years (Fig. 8a) and −1.42 in cold-spring years (Fig. 8b). Based on these statistical results, Rs dominated Re when the EVI was less than 0.48 in the normal years, whereas in the cold-spring years, the Rs constituted a larger proportion when the EVI was less than 0.41.

**Figure 9:**
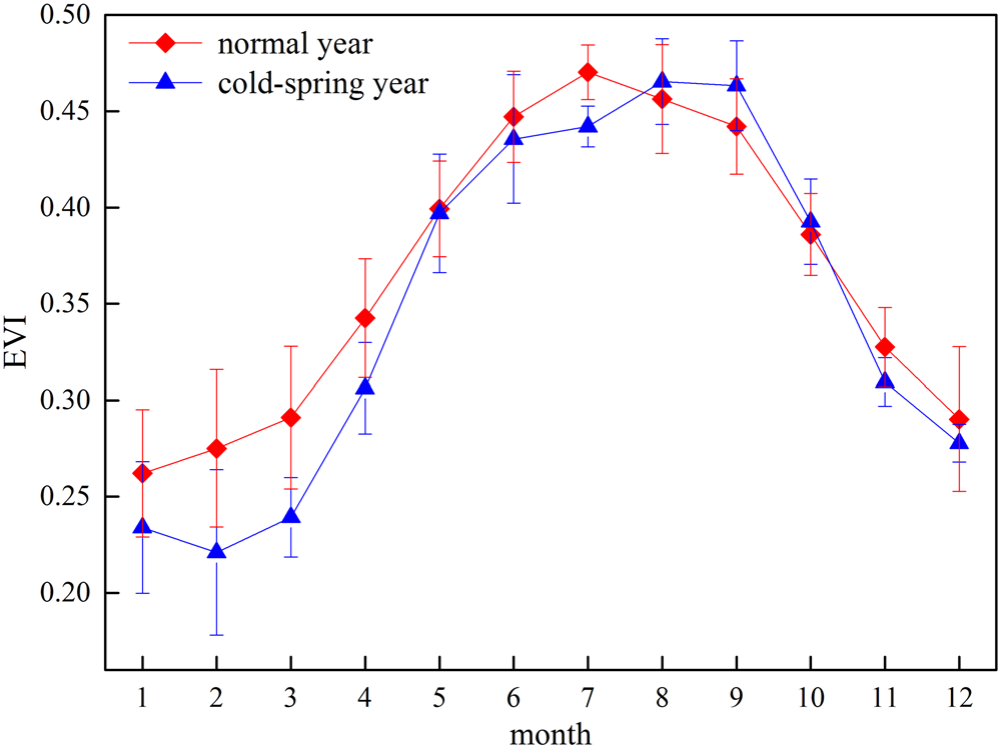
Multi-year averaged monthly MODIS EVI for the coniferous plantation region around the QYZ site. The blue line indicates years that experienced cold springs, and the red line indicates normal years.

In addition, the EVI showed great differences in normal and cold-spring years (Fig. 9). Firstly, the EVI during January to April was much lower in clod-spring years than that in normal years. Secondly, the cold-spring postponed the peak time of EVI, which showed up in August and September instead of July in normal years. These phenomena indicated the great differences of ecosystem functions between normal and cold-spring years, which agreed well with some previous researches^12, 41^.

In normal years, the EVI was lower than 0.48 in autumn (Fig. 9). Combing the results of Fig. 8 as mentioned above, the Rs was generally found to account for a larger proportion of Re in normal years. As the Rs is positively related to precipitation^70^, the increases in autumn precipitation would increase the total annual Re. In contrast, Re in cold-spring years showed a decrease trend with increasing autumn precipitation, possibly due to the decreasing effects of precipitation on plant respiration. In the cold-spring years, the plant respiration constituted over 50% of the Re in the autumn according to Figs 8 and 9. Therefore, autumn precipitation might have an opposite effect on Re in the years with cold springs.

However, the ecosystem responses to the precipitation are complicated and difficult to quantify. To demonstrate the underlying mechanisms, this study explored the lag effects of precipitation on GPP and the divergent effects on Re in cold-spring and normal years. Indeed, predicted climate change may include more frequent and severe dry seasons in response to global warming^72^ and more frequent El Niño episodes^73^. The results reported here indicate that the effects of precipitation must be considered to better constrain the uncertainties associated with estimation under that projected scenario.

## Materials and Methods

### Site description

This study was conducted in a subtropical coniferous plantation (26°44′29″N, 115°03′29″E, at 102 m elevation above sea level) at Qianyanzhou Ecological Research Station (QYZ), located in a typical red soil hilly region in south China, with a subtropical monsoon climate. The prevailing wind direction of this climate regime is north-northwest in the winter and south-southeast in the summer. The coniferous trees were planted in approximately 1985. The dominated species are Masson pine (*Pinus massoniana* Lamb.), Slash pine (*Pinus elliottii* Englem.) and Chinese fir (*Cunninghamia lanceolata* Hook.), with a tree density of approximately 1460 stems ha^−1^ and a total biomass of 106 t ha^−1^. The red soil is weathered from red sand rock, and the soil texture is composed of 2.0–0.05 mm (17%), 0.05–0.002 mm (68%) and <0.002 mm (15%). Further details of the QYZ site can be found in refs 31 and 70.

Based on meteorological observations from 1989 to 2014 at QYZ, the mean annual temperature is 18.0 °C, and the mean annual precipitation is 1504.6 mm. In February, daily air temperature increases gradually from the annual minimum (on average, 4.66 °C) to greater than 10 °C. For this Ta sensitive period, annual cumulative air temperature ( > 5 °C) varies greatly, between 41.7 and 217.3 °C (SD 64.3 °C). The annual precipitation varies between 945 and 2144 mm (SD 12 mm), approximately 24%, 41%, 23% and 12% of which occurs in the four quarters of the year in turn. Twelve years (2003–2014) of flux data were observed with the eddy covariance method in a subtropical forest plantation at QYZ and used for this study.

### Observation and instrumentation

The eddy covariance flux observation system was established in a subtropical coniferous plantation at QYZ site in 2002. The above-canopy flux was measured at a height of 23.6 m by instruments loaded on a ventilated tower. The wind velocity was detected by a 3-D sonic anemometer (Model CSAT3, Campbell Scientific Inc., Logan, UT, USA), and the variations in CO_2_ and water vapor concentration were measured using an open-path CO_2_/H_2_O analyzer (Model LI-7500, Li-cor Inc., Lincoln, NE, USA). All signals were sampled at a frequency of 10 Hz, and the CO_2_ and H_2_O fluxes were calculated and recorded at 30 min intervals by a CR5000 datalogger (Campbell Scientific Inc.).

Air temperature and relative humidity sensors (Model HMP45C, Campbell Scientific Inc.) were mounted under ventilated shields at heights of 23.6 m and 39.6 m above the ground, respectively. Soil temperature and soil water content were measured at a depth of 5 cm with thermocouples (105 T and 107-L, Campbell Scientific Inc.) and TDR probes (Model CS615-L, Campbell Scientific Inc.), respectively. Radiation measurements were completed using a four-component net radiometer (Model CNR-1, Kipp & Zonen, Delft, ZuidHolland Netherlands), a pyranometer (Model CM11, Kipp & Zonen) and a quantum sensor of photosynthetically active radiation (Model LI190SB, Licor Inc.). Rainfall was monitored with a rain gauge (Model 52203, RM Young Inc., Traverse, MI, USA). Meteorological variables were sampled at 1 Hz, and 30 min average data were recorded with three CR10X dataloggers and a CR23X datalogger with a 25-channel solid-state multiplexer (Campbell Scientific Inc.).

### Flux correction and gap filling

This study adopted the methods of calculating and correcting carbon dioxide fluxes in Wen *et al*.^17, 31^. The CO_2_ fluxes were calculated every 30 minutes from the 10 Hz raw data. Processing of the flux data was performed using routine methods, including three-dimensional rotation^74^, the Webb, Pearman and Leuning correction for the effects of air density fluctuations (WPL correction)^75^, storage calculations and spurious data removal^8, 31^. Spurious data caused by rainfall, water condensation or system failure were removed from the dataset. To avoid the possible underestimation of the fluxes under stable conditions at night, nighttime data (solar elevation angle < 0) were excluded when the friction velocity (u_*_) was less than the relevant thresholds, which were identified based on the researches of Reichstein *et al*.^76^. The threshold values of u_*_ ranged from 0.16 to 0.22 m s^−1^, with an average value of 0.20 m s^−1^ for the years from 2003 to 2014. Any data gaps in meteorological variables were filled using the mean diurnal variation method^77^. The linear fitting, nonlinear fitting and mean diurnal variations were used to fill missing data points and to replace spurious data^43^. Further details of data processing are presented in ChinaFLUX^17, 43, 74^.

### Data analysis

The carbon fluxes and their corresponding main climatic factors, including direct solar radiation (DR), air temperature (Ta), precipitation (PP), vapor pressure deficit (VPD) and soil water content (SWC), were integrated at the month and annual scales to represent seasonal and inter-annual variation. Their anomalies were also calculated to indicate their deviations from normal levels. In previous studies, this subtropical coniferous plantation was temperature sensitive in early spring^8^. Therefore, the averaged early spring (January to March) temperatures were calculated. In addition, for the purpose of our study, the cumulative autumn precipitation values (September to November) were calculated.

The linear relationships between annual carbon flux and precipitation were calculated to determine the sensitivity of carbon flux. In addition, the linear relation between annual carbon flux and autumn precipitation in the same year and in the previous year were estimated to examine the sensitivity of this ecosystem to autumn precipitation.

### Lag effects of precipitation on GPP/Re

To identify the relationship between autumn precipitation and GPP/Re, we analyzed their correlation at the annual scale, placing emphasis on the time lag effects between the autumn precipitation and GPP/Re.

For this purpose, annual cumulative precipitation and GPP/Re were calculated. Multiple “yearly” statistics (approximately 130 values from the twelve years) were obtained for 12-month intervals, shifting them one month at a time^33, 41^. To investigate the lag of the GPP/Re response to the climatic factors, we shifted the climatic series backward one month at a time (up to twelve months) to calculate the correlations between climate drivers and GPP/Re. Student’s *t*–tests were applied to verify the statistical significance of the correlation coefficients^28, 33^.
